# Application of loop-mediated isothermal amplification and next-generation sequencing in the diagnosis of maternal tuberculosis with multiple negative tests: A case report

**DOI:** 10.1097/MD.0000000000027387

**Published:** 2021-10-01

**Authors:** Hongwei Li, Qian Wu, Jinmiao Hu, Liting Feng, Qi Wu, Hongzhi Yu, Li Li, Xinhui Li

**Affiliations:** a Department of Respiratory and Critical Care Medicine, Haihe Hospital, Tianjin University, Tianjin, China; b Guangzhou Sagene Biotech Co., Ltd., Guangzhou, China.

**Keywords:** diagnosis, loop-mediated isothermal amplification, maternal tuberculosis, next-generation sequencing

## Abstract

**Rationale:**

Tuberculosis (TB) is one of the top 10 causes of death worldwide and is the leading infectious cause of death. The incidence of TB, especially active TB, is increased in pregnant and postpartum women. Newborns can be infected with TB from their mothers through several routes. Diagnosis of TB in pregnant women and infants is difficult. Here, we report the simultaneous postdelivery diagnosis of TB in a mother and infant pair.

**Patient concerns:**

A 28-year-old woman presented with a sudden onset of convulsions, loss of consciousness, coughing, fever, and breathing difficulty. Her 18-day-old infant daughter developed cough and wheezing.

**Diagnosis:**

The mother's chest computed tomography showed diffuse interstitial changes and both lungs’ exudation. Enhanced cranial magnetic resonance imaging showed scattered nodular intracranial lesions. A tuberculin skin test and an interferon-gamma release assay were negative. Xpert MTB/RIF (Xpert) testing and acid-fast bacilli smear of bronchoalveolar lavage (BAL) fluid of the mother were negative. Loop-mediated isothermal amplification of BAL fluid was positive for *Mycobacterium tuberculosis*, and next-generation sequencing confirmed the diagnosis of TB. A biopsy specimen also showed characteristic TB findings. The mother was diagnosed with TB and TB encephalitis. The infant's BAL fluid was positive for acid-fast bacilli and Xpert and, therefore, was diagnosed with TB.

**Interventions:**

The mother was treated with rifampicin and isoniazid for 9 months, ethambutol and pyrazinamide for 3 months, and prednisolone acetate for 8 weeks. The infant received ventilator-assisted ventilation for 10 days and anti-tuberculous therapy for 11 months.

**Outcomes:**

After anti-tuberculous therapy, the mother and infant both gradually recovered. The mother's chest computed tomography showed significant recovery 9 months after discharge. The infant developed normally during the 11-month follow-up.

**Lessons:**

This mother-child case pair highlights the value of loop-mediated isothermal amplification and next-generation sequencing as new diagnostic technologies for diagnosing TB in patients with multiple negative tests.

## 1 Introduction

According to the 2020 World Health Organization report, tuberculosis (TB) is one of the top 10 causes of death worldwide and is the leading infectious cause of death.^[1]^ The incidence of TB, especially active TB, is significantly increased in pregnant and postpartum women.^[2,3]^ Newborns can be infected with TB from their mothers through several routes, such as the placenta or blood, via inhalation of infected amniotic fluid, during childbirth (in maternal genital tract infection), or from postpartum contact with the mother.^[4]^ Symptoms such as fatigue and loss of appetite caused by TB in pregnant women are easily confused with the symptoms of pregnancy itself, leading to delayed diagnosis and treatment.^[5]^ The tuberculin skin test (TST) is a valuable pregnancy screening test, and a shielded chest x-ray of suspected TB patients is necessary.^[6]^ Laboratory investigations, including smear microscopy, Xpert MTB/RIF (Xpert), and mycobacterial culture, should also be considered.^[7]^

Although many diagnostic methods have emerged, the diagnosis of TB in pregnant women and children remains difficult.^[8]^ Herein, we report a case of simultaneous diagnosis of TB in a mother and infant pair during the neonatal period, and discuss the application of new diagnostic technology, including loop-mediated isothermal amplification (LAMP) and next-generation sequencing (NGS).

## 2 Case report

**Figure 1:**
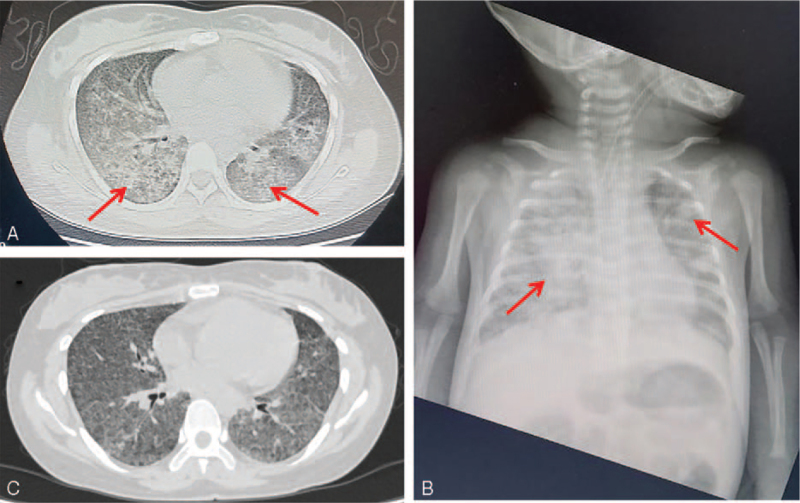
(A) Chest computed tomography (CT) of the patient (mother) revealing diffuse interstitial changes and both lungs’ exudation (red arrow). (B) The chest x-ray of the patient's infant daughter showing multiple patchy shadows in both lungs (red arrow). (C) Chest CT of the patient (mother) after 9 months of anti-tuberculous therapy.

A 28-year-old woman was admitted to our hospital due to a sudden onset of convulsions, which had lasted for 3 hours and was accompanied by loss of consciousness for about 1 minute without headache or vomiting. She had been diagnosed with syphilis 1 year before presentation. In the month that preceded her hospitalization, the patient had experienced persistent coughing. Four days before hospitalization, she had intermittent fever (maximum temperature: 39.4°C) and breathing difficulty. She gave birth to a baby girl 18 days before presenting to the hospital. Physical examination revealed tachycardia. Without oxygen inhalation, her SpO_2_ was low at 75%, and the oxygenation index was 240 mm Hg. After 3 L/min of oxygen inhalation via a nasal catheter, her SpO_2_ rose to 90%. Chest computed tomography showed diffuse interstitial changes, and both lungs’ exudation (Fig. 1A). Laboratory tests showed elevated C-reactive protein, leukocyte, and neutrophil levels in the peripheral blood (28.06 mg/L, 7.46 × 10^9^ cells/L, and 80.4%, respectively). D-dimer levels were significantly increased (>10,000 ng/mL). A non-treponemal test (the toluidine red unheated serum test) revealed titers of 1:4, which was negative, and a treponemal test (*Treponema pallidum* particle agglutination) was positive. Her human immunodeficiency virus antigen and antibody test results were negative. Lumbar puncture was performed, and the obtained cerebrospinal fluid was colorless and transparent, the intracranial pressure was 80 mmH_2_O, and cerebrospinal fluid routine and biochemistry test results were normal. Enhanced cranial magnetic resonance imaging showed scattered nodular intracranial lesions. She was suspected of having miliary TB, and further investigations were performed, including a TST and interferon-gamma release assays (IGRAs), which were negative. The patient received empirical antibiotic therapy with piperacillin/tazobactam and moxifloxacin, and antiviral therapy with oseltamivir, but she did not respond to treatment. In addition, the infant developed cough and wheezing, and was admitted to the neonatology department. The infant's chest x-ray showed multiple patchy shadows in both the lungs (Fig. 1B). The infant received penicillin antibiotic therapy, but her clinical condition did not improve. On the second day after admission, she received ventilator-assisted ventilation because of respiratory failure.

**Figure 2:**
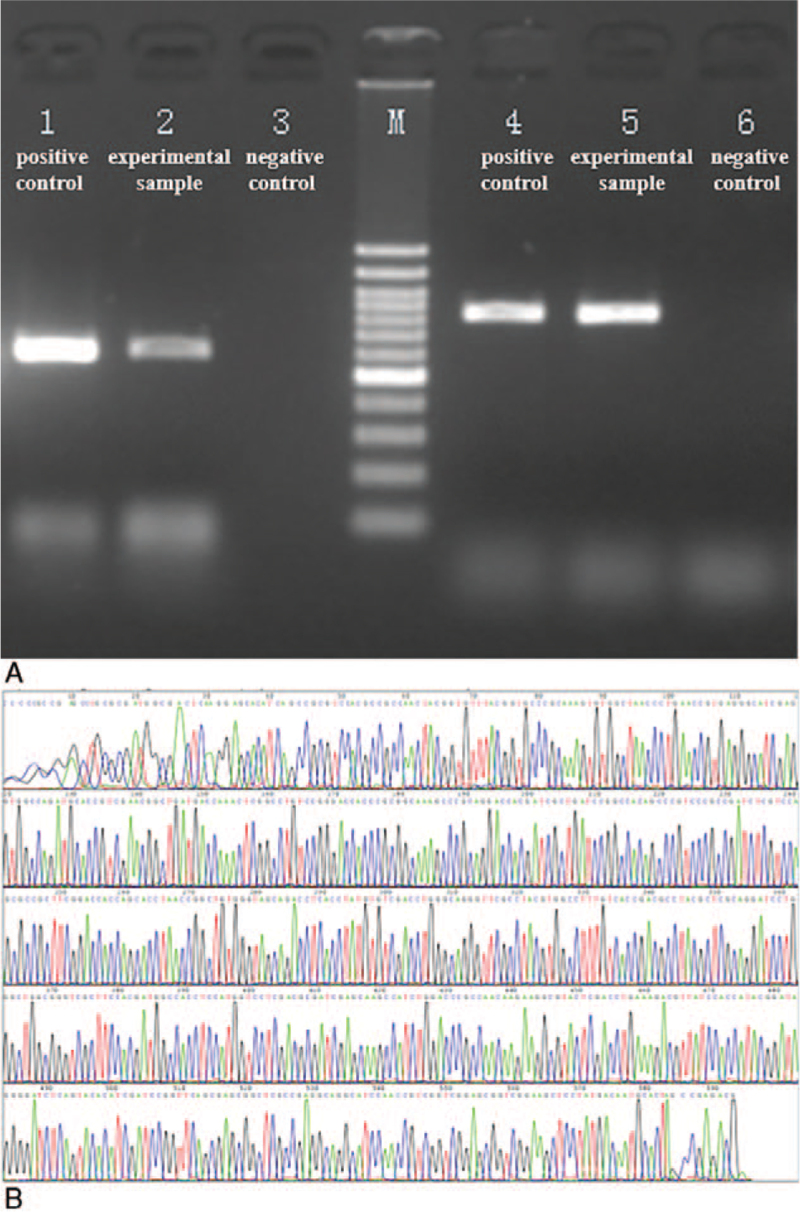
(A) Electrophoretogram of polymerase reaction chain products of S-Reagent verification systems (Lanes 1–3 include targeted amplification results using Mycobacterium, and lanes 4–6 include targeted amplification results for *Mycobacterium tuberculosis*, among which lanes 1 and 4 are positive control samples, lanes 2 and 5 are experimental samples, and lanes 3 and 6 are negative control samples). (B) Graph of peaks produced via Sanger method. The results were compared to sequences in the National Center for Biotechnology Information database and confirmed *Mycobacterium tuberculosis* diagnosis.

Bronchoscopy was performed on the mother 1 week after admission to clarify her diagnosis. The bronchoalveolar lavage (BAL) fluid was analyzed using Xpert and smear microscopy for acid-fast bacilli, which both revealed negative results. However, testing of BAL fluid using LAMP revealed positive results. Samples of BAL fluid were also sent for NGS (RDP-seq, Sagene, Guangzhou). In total, 20,906,582 reads were obtained, with 120, 50, 6, and 1 sequencing reads mapped to *Prevotella melaninogenica*, *Enterococcus faecium*, *Veillonella parvula*, and *Mycobacterium* sp., respectively. The relative abundance of the 4 bacteria was 67.80%, 28.25%, 3.39% and 0.56%, respectively, calculated according to the percent of the microorganism type. Polymerase reaction chain and Sanger sequencing for *Mycobacterium tuberculosis* (MTB) were performed subsequently using an S-Reagent verification system (Sagene, Guangzhou). Polymerase reaction chain products were electrophoresed (Fig. 2A), sequenced using Sanger method (Fig. 2B), and compared to sequences in the National Center for Biotechnology Information database. A comparison of results confirmed the MTB diagnosis.

**Figure 3:**
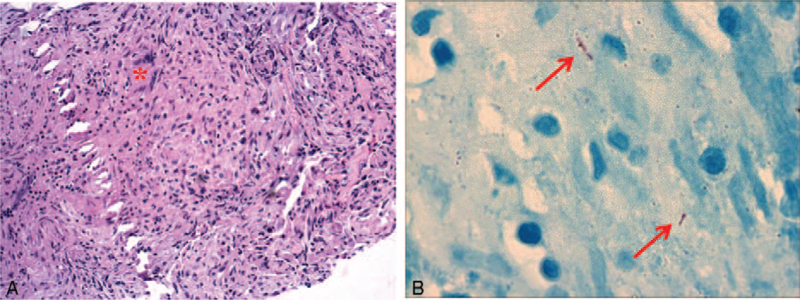
(A) Histopathologic examination of the right upper posterior bronchus biopsy specimen showing epithelioid granulomas and multinucleated giant cells (red asterisk) (hematoxylin-eosin staining, ×20). (B) Acid-fast bacilli in a Ziehl-Neelsen-stained biopsy specimen (red arrow).

The patient (mother) underwent repeat bronchoscopy 3 weeks after admission once her symptoms had improved. A bronchoscopic biopsy specimen taken from the right upper posterior bronchus was used to confirm the TB diagnosis. Histopathologic examination showing epithelioid granuloma and multinucleated giant cells in the specimen was consistent with TB (Fig. 3A). The biopsy specimen visualized via microscopy was positive for acid-fast bacilli using Ziehl-Neelsen staining (Fig. 3b), and positive CD68 was observed on immunohistochemical staining. Therefore, the mother was diagnosed with TB and TB encephalitis. The infant's BAL fluid was also positive for acid-fast bacilli and Xpert; therefore, she was also diagnosed with TB.

The patient (mother) was treated with rifampicin (450 mg daily) and isoniazid (300 mg daily) for 9 months, ethambutol (750 mg daily) and pyrazinamide (500 mg 3 times per day) for 3 months, and prednisolone acetate (20 mg twice a day, reduced by 5 mg per week) for 8 weeks. After administering anti-tuberculous therapy, the patient's symptoms gradually improved. Her convulsions, dyspnea, fever, and cough resolved. Further, chest computed tomography showed significant recovery after 9 months of anti-tuberculous therapy (Fig. 1C). The infant also received anti-tuberculous therapy of rifampicin (37.5 mg daily) and isoniazid (37.5 mg daily) for 11 months, and ethambutol (75 mg daily) and pyrazinamide (100 mg daily) for 3 months. She was successfully removed from the ventilator after 10 days and was discharged from the hospital after 23 days treatment. The infant had no further respiratory symptoms and developed normally during the 11-month follow-up.

The study was approved by the ethics review committee of Haihe Hospital, Tianjin University.

## 3 Discussion

As the mother had a history of syphilis, we considered syphilis with pulmonary involvement in the differential diagnosis. Her toluidine red unheated serum test result was negative and *Treponema pallidum* particle agglutination test result was positive, which suggested that the patient had a history of syphilis rather than active syphilis.^[9]^ We also found *P melaninogenica*, *E faecium*, and *V parvula* in the BAL fluid on NGS. Since these are common in the oral and intestinal flora, they were not considered to be the pathogenic bacteria in the patient.

Chronic cough in mothers during the perinatal period should be taken seriously because 60% of antenatal women diagnosed with TB develop cough symptoms lasting longer than 2 weeks, and 30% develop fever or night sweats.^[10]^ Unusually, the mother tested negative via TST, IGRAs, sputum smear, and culture. However, MTB was confirmed by LAMP and NGS of the BAL fluid. False-negative TST and IGRAs may be associated with miliary TB and TB meningitis.^[11,12]^ Perinatal TB is difficult to diagnose because the pro-inflammatory response of Th1 cells during pregnancy is suppressed, which suppresses TB symptoms.^[13]^ Immune reconstitution of Th1 postdelivery may lead to aggravation of TB symptoms.^[14]^

Rapid and accurate TB diagnosis is important because it is vital to patients’ immediate treatment to minimize the risk of transmission of this airborne disease to others in the community. In recent years, molecular diagnostic techniques based on TB gene detection and nucleic acid amplification have developed rapidly, and the representative detection techniques include Xpert and LAMP.^[15]^ These technological advances have led to breakthroughs in the early detection of TB compared to traditional sputum smear tests. As in our case, the TST and IGRA results were negative, as were sputum smear tests for TB. Further Xpert analysis of BAL fluid was also negative. However, the initially strong suspicion of TB was confirmed via BAL fluid LAMP.

LAMP is superior to Xpert in diagnosing smear-negative TB and is also advantageous because its reagent is less costly. The World Health Organization recommends LAMP as a replacement for microscopy to diagnose pulmonary TB in adults with TB signs and symptoms.^[16]^ NGS is also an effective method for the rapid diagnosis of TB.^[17]^ It can be used for the genetic analysis of drug-resistant TB and susceptible strains.^[18]^ Mixed infections are also identified.

Maternal and neonatal deaths have always been health care and indicators focused upon by governments globally. In this case, the conditions of both the mother and infant were critical and complex. The doctors carefully analyzed the patient's laboratory results and combined them with disease history and clinical experience. Finally, the cause of the disease was determined to address and alleviate symptoms. LAMP and NGS, as new diagnostic technologies, are potentially useful for facilitating the diagnosis of TB in patients with multiple negative tests.

## Acknowledgment

We would like to thank Guangzhou Sagene Biotechnology Co., Ltd. for its detection of *Mycobacterium tuberculosis* and their help in the clinical diagnosis.

## Author contributions

**Conceptualization:** Hongzhi Yu.

**Data curation:** Hongwei Li, Jinmiao Hu, Liting Feng.

**Formal analysis:** Liting Feng, Li Li.

**Methodology:** Xinhui Li.

**Resources:** Li Li.

**Supervision:** Qi Wu.

**Visualization:** Qian Wu.

**Writing – original draft:** Hongwei Li, Qian Wu.

**Writing – review & editing:** Hongzhi Yu.
